# Allochthonous carbon is a major regulator to bacterial growth and community composition in subarctic freshwaters

**DOI:** 10.1038/srep34456

**Published:** 2016-09-30

**Authors:** Toni Roiha, Sari Peura, Mathieu Cusson, Milla Rautio

**Affiliations:** 1 Department of Biological and Environmental Science, University of Jyväskylä, Jyväskylä, Finland; 2 Département des sciences fondamentales, Université du Québec à Chicoutimi, Chicoutimi, Quebec, Canada; 3 Department of Ecology and Genetics, Limnology, Uppsala University, Uppsala, Sweden; 4 Centre for Northern Studies (CEN), Laval University, Quebec City, Quebec, Canada.; 5 Group for Interuniversity Research in Limnology and aquatic environment (GRIL), University of Montreal, Montreal, Quebec, Canada.

## Abstract

In the subarctic region, climate warming and permafrost thaw are leading to emergence of ponds and to an increase in mobility of catchment carbon. As carbon of terrestrial origin is increasing in subarctic freshwaters the resource pool supporting their microbial communities and metabolism is changing, with consequences to overall aquatic productivity. By sampling different subarctic water bodies for a one complete year we show how terrestrial and algal carbon compounds vary in a range of freshwaters and how differential organic carbon quality is linked to bacterial metabolism and community composition. We show that terrestrial drainage and associated nutrients supported higher bacterial growth in ponds and river mouths that were influenced by fresh terrestrial carbon than in large lakes with carbon from algal production. Bacterial diversity, however, was lower at sites influenced by terrestrial carbon inputs. Bacterial community composition was highly variable among different water bodies and especially influenced by concentrations of dissolved organic carbon (DOC), fulvic acids, proteins and nutrients. Furthermore, a distinct preference was found for terrestrial vs. algal carbon among certain bacterial tribes. The results highlight the contribution of the numerous ponds to cycling of terrestrial carbon in the changing subarctic and arctic regions.

Dissolved organic matter (DOM) in the surface waters is a complex mixture of humic substances, carbohydrates, carboxylic acids, amino acids and nutrients. These compounds originate from terrestrial and aquatic production, make about 90% of the total carbon pool in the water column and are a major energy source for the aquatic food webs^1^. The compounds originating from aquatic primary production are usually considered to have better quality and, hence, be more biolabile than compounds with terrestrial origin. However, several recent studies have shown that some terrestrially-derived carbon compounds can promote aquatic production^2^,^3^. The ratio between terrestrial and algal DOM still has an important role in determining carbon cycling in a lake, including the productivity of algal and microbial biomasses^4^ and greenhouse gas (GHG) releases^5^. Lakes with high reliance to terrestrial DOM tend to be less productive and net heterotrophic, i.e. CO_2_ and CH_4_ sources to the atmosphere^6^,^7^ while lakes in which the food web is largely based on carbon of autochthonous origin are characterised with high levels of primary productivity making the lakes net autotrophic, i.e. CO_2_ sinks^8^. For a long time most arctic lakes were, due to the sparse catchment vegetation, considered to be only marginally influenced by allochthonous matter inputs^8^. However, the on-going climate warming is mobilizing terrestrial carbon pools and increasing the allochthonous carbon inputs from the catchment to surface waters worldwide^9^,^10^,^11^. Subsequently, the subarctic and arctic regions impacted by permafrost thawing are experiencing the mobilization of the Earth’s largest pool of organic carbon^12^ and nutrients^13^. There are, however, still few comprehensive studies examining organic carbon pools and associated microbial metabolism and bacterial communities in northern freshwaters although these environments constitute an integral component in the catchment-water-climate continuum.

Large compositional changes take place from the moment terrestrial carbon arrives to a lake and when it is mixed with algal DOM exudates and transferred to new compounds through photochemical and microbial processes^14^,^15^,^16^. Proportions of different fractions of DOM also vary across lakes and within individual water bodies due to physical factors in the environment^17^,^18^. For example, in small ponds soil carbon enters the water body by runoff along the land-water interface, while most of the terrestrial organic matter arriving to lakes is loaded through the river inlets^19^. This generates a spatial pattern at the landscape level in the availability of terrestrial C in morphologically different water bodies, although it has received little attention. Morphometric differences among different freshwaters are especially pronounced and changing in the northern environments where thawing permafrost is at an accelerated rate promoting the formation of shallow ponds. They have become increasingly abundant in the circumpolar north, representing up to 90% of all freshwater in some regions^12^. Growing attention has been given to these new ponds after recognizing the cumulative effect of their high abundance and greenhouse gas (GHG) emissions on global warming^20^,^21^ while little is known about organic carbon differences among northern ponds, lakes and rivers. Morphometric differences between water bodies influence photo exposure, residence time, velocity and primary production, all of which contribute to defining DOM. The water column depth plays a critical role as photochemical processes in shallow euphotic zones make DOM more bioavailable to bacteria compared to DOM in the dark^16^. The efficiency of DOM transformations also decreases as organic matter ages^22^ making the sites that are physically close to the terrestrial source first and mostly influenced by allochthonous carbon. For instance, in large lakes the long distance to the shore dilutes the influence of terrestrial DOM to food webs^23^.

Since the DOM composition changes depending on the catchment-lake coupling and among morphologically different lakes^24^, we can reasonably expect that heterogeneity in the various C sources among ponds, lakes and rivers may lead to variability in the composition of organisms capable of using different carbon sources. Variable microbial communities mediate the immediate carbon sequestration processes of the arriving fresh allochthonous carbon, are involved in its cycling and uptake to the food web, and contribute to respiration that removes terrestrial carbon from the water body via atmospheric emissions. Proteins and amino acids from algal exudates are being readily utilized by bacteria^25^, while the recalcitrant compounds in humic substances, such as lignin, can be degraded only by more specialized groups^26^. Still, water masses dominated by recalcitrant humic compounds may facilitate high rates of microbial metabolism in favourable conditions. The evidence of a priming effect of high quality carbon in freshwaters is still conflicting^27^, but algal exudates have been suggested to act as a primer for the consumption of the recalcitrant carbon pool^28^. Such complex biochemical processes need to be considered when establishing causalities between DOM composition and bacterial metabolism, and may largely vary among morphologically different water bodies. Nevertheless, the variation in carbon quality shapes the bacteria residing in lakes and it has been shown that bacterial community composition (BCC) and metabolism are linked to the source^29^,^30^,^31^,^32^ and quality of the carbon found in a lake^33^,^34^,^35^. Further, it has been shown that the composition of bacterial community plays a significant role in the rate of carbon mineralization^36^. While bacteria are processing DOM, some compounds are produced, while others get degraded^14^,^15^. Thus, bacteria are influenced by the DOM milieu, but also contribute to defining the quality and quantity of carbon in lakes.

In this paper, we investigate the importance of terrestrially-derived DOM (t-DOM) to bacteria in the changing subarctic region. Our objective is to estimate how climate-warming induced increase in t-DOM is influencing subarctic aquatic microbiome. Our expectation was that the relative proportion of t-DOM in subarctic freshwaters is the greatest in small and shallow water bodies that are the dominant lake type in the subarctic and arctic regions, and increasing in numbers^37^, while pelagic waters of large lakes would be less influenced by t-DOM and would be characterised by the DOM fraction that originates from within lake primary production. Further, we expected that differences in the amount of t-DOM and algal exudates would lead to different metabolic rates, measured as bacterial production, respiration and growth efficiency. We also anticipated the bacterial community composition to be adapted to different substrata and, thus, to vary along the range of t-DOM exposure (i.e. among morphologically different types of water bodies). To study these questions, we sampled lake inlets representing sites that should be influenced by fresh allochthonous carbon arriving to lakes, outlets of large lakes i.e. sites that integrate carbon from in-lake pelagic and near-shore benthic algal production, and ponds that are exposed to high loads of t-DOM due to their small shoreline-area ratio and at the same time to high overall primary production due to the illuminated total water column and benthos^38^, and that should contain carbon with a mixed signature of terrestrial and algal compounds. We further discuss the potential impact of the increasing number of shallow ponds on the carbon pools and microbiome of subarctic and arctic freshwater ecosystems. All sites were monitored in five different seasons and bacterial metabolism and community composition were analyzed in relation to DOM quality (carbon compounds, spectrophotometric properties, nutrients).

## Materials and Methods

### Study site and sampling

We sampled lake inlets, lake outlets and ponds in the Kilpisjärvi region, subarctic Finnish Lapland (69 ⚬N, 20 ⚬E), with catchments extending to Sweden and Norway, for one complete year and collected a data set that was based on 45 site visits. The sites were located at an altitude between 472 and 850 m a.s.l. in the subarctic landscape where treeline, mainly mountain birch (*Betula pubescens* subsp. *czerepanovii*), is at 600 m a.s.l. Apart from the birch, vegetation mainly consists of low dwarf shrubs, mosses, grasses and sedges, with a low occurrence of wetlands (about 10%). The ponds sampled (11, 12 and 15, as in Rautio^39^) had a mean depth of 4 m, area of 0.9 ha and a catchment of 42 ha. The inlets (Lakes Saanajärvi, Tsahkaljärvi and Kilpisjärvi) collected runoff along a mean distance of 2100 m and the outlets integrated water from lakes with a mean depth of 35 m and a lake area of 1294 ha. More detailed characterization of the individual water bodies presented in Supplementary Table 1. Each site was sampled five times in 2011; in February (winter), in early May (spring), in mid-June just after the ice break up (ice break up), in late July (summer) and in early October (fall). Ponds were sampled in the middle of the pond and the lakes were sampled from near the inlets and outlets. Samples were collected with a 2 L Limnos water sampler (Limnos ltd, Turku, Finland) as integrated samples from the first meter of the water column. Water temperature was measured in the field with YSI Professional Plus (Yellow Springs, OH, USA). Total phosphorus (TP) and total nitrogen (TN) concentrations were analysed from sieved (50 μm) water using standard methods (http://www.sfs.fi/). For the determination of chlorophyll a (Chl-*a*) concentrations, 1–2 L was filtered onto GF/F filters. Samples were collected in duplicate and stored at −80 °C until fluorometric analysis was carried out according to Nusch^40^. Dissolved organic carbon (DOC) concentration was analysed from water filtered through 0.2 μm prerinsed cellulose acetate filter using Shimadzu TOC-5000A carbon analyser.

### DOM quality

A set of carbon quality indicators was measured using spectrophotometric and spectrofluorometric methods. All the measurements were carried out for water that had been filtered through a 0.2 μm prerinsed cellulose acetate filter and stored in the dark at +4 °C. Absorption coefficient at 320 nm (a320), specific UV-absorbance index (SUVA) and the spectral slope (S289) were measured in a dual-beam mode with Cary 100 UV-Vis spectrophotometer (Agilent) using a 10-cm quartz cuvette. Baseline correction was done with MilliQ-water. Absorption coefficient at 320 nm (a_320_) was measured as an indicator of total concentration of coloured dissolved organic carbon (CDOM_320_). Values were calculated from absorbance measurements (A_λ_) at 320 using a_λ_ = 2.303/L × A, where L is the length of the cuvette in meters^41^. SUVA, which is an indicator of the share of terrestrially derived organic carbon^18^,^42^, was calculated from DOC-normalized absorbance at the wavelength 254 nm with higher values indicating a higher share of terrestrial carbon compounds in the sample^43^. S289, indicating the amount of carbon compounds related to lignin-free algal production^44^, was calculated from the spectrophotometric measurements between 279–299 nm. Slopes were calculated over 20 nm intervals with a 1 nm step (i.e., 250–269, 251–270, etc.). The resulting set of spectral slopes was plotted by center wavelengths. Calculations were performed in open-source software package SciLab 4.15. The individual spectral slope S289 was used to evaluate the amount of autochthonous compounds related to autochthonous production. Algal derived lignin-free carbon has its maximum absorbance close to 289 nm^44^,^45^,^46^ thus the higher the S289 values the bigger is the share of carbon compounds from autochthonous production. There are some environmental factors that could have compromised the fluorometric measurements, most importantly iron and pH. According to previous measurements of the lakes in the area the iron concentration is low (mean of 37 lakes 0.24 mg L^−1^)^47^ and not likely to cause a bias. Also, the pH was stable within the samples (6.5 ± 0.5) and should not interfere with the measurements. Thus, we are confident that our measurements were reliable and showing the true variation in carbon quality.

Composition of different humic, fulvic and protein-like carbon compounds was identified with excitation-emission matrixes (EEM) using a spectrofluorometer Cary eclipse (Agilent). Those were measured across excitation (220–450 nm) and emission (240–600 nm) wavelengths with 5 and 2 nm increments, respectively. EEMs were corrected for inner filter effect^48^, machine specific biases, background scattering^49^ and were standardized to Raman units (R.U.)^50^. Raman and Rayleigh scattering were removed using the DOMfluor 1.7 toolbox in MATLAB 2008b (MathWorks, Natick, MA, USA) as recommended by Stedmon and Bro^51^. The obtained EEMs were inserted to the parallel factor analysis (PARAFAC) model based on samples collected from >100 lakes from boreal, subarctic and arctic lakes from Finland, Canada and Greenland (data not shown). The model was used to identify and calculate intensities of all main carbon components in the samples. Five different components (C1-C4, C6) identified from the EEMs were highly correlated with each other (correlation coefficients for all pairs >0.87, p < 0.0001) and were pooled for the analyses as terrestrial humic-like compounds, while the component C5 was considered as a fulvic acid and the component C7 as a protein, according to Fellman *et al.*^52^. The compounds C1-C4 and C6 are widespread terrestrial humic-like components originating e.g. from forest streams and wetlands^52^ (and references therein). The C5 compound has been associated with irradiated DOM that has been microbially degraded^53^. C7 resembles amino acid-like tryptophan found commonly in different freshwater environments^52^.

### Bacterial metabolism analyses

Bacteria production (BP) was measured using ^3^H-leucine (specific activity 73 Ci mmol^−1^) incorporation with a centrifugation method^54^. Incubations were started within 2–6 hours after sampling using a leucine concentration of 30 nM and incubation time of 3 h according to the saturation curves in Roiha *et al.*^35^. Incubations were conducted in the dark in 6.4 ± 0.5 °C temperature. The difference to the *in-situ* field temperatures was 5.1 ± 2.1 °C. TCA was added to terminate incubation (TCA; 5% final concentration) after which the samples were stored at −20 °C until centrifuging and radioassaying according to Smith and Azam^54^. Bacterial respiration (BR) was measured as oxygen (O_2_) consumption using fibre-optic O_2_ mini-sensors (Fibox 3, PreSens Precision Sensing GmbH, Regensburg, Germany)^55^. Filtered (3 μm pore size) water samples were incubated in headspace-free 500 ml Erlenmeyer vials closed with airtight silicone stopper. Samples were incubated as above but in a water bath to further reduce temperature variability, which could interfere with O_2_ sensor reading. The incubations were let to stabilize for a few hours before the first sensor reading. Over the first five days O_2_ concentrations were measured 1–2 times a day while the last measurement was taken at the beginning of the next sampling (total incubation time 4–6 weeks). BR rates were calculated from the linear slope of O_2_ consumption that was converted to carbon units using respiratory quotient (RQ) of 1.0 as in Berggren *et al.*^56^. To estimate actual bacterial metabolism in the sampled sites, the BP and BR values were corrected for *in-situ* temperatures with Q_10_ values according to Berggren *et al.*^57^. Such corrections were not applied when the aim was to measure temperature-independent bacterial control. Bacterial growth efficiency (BGE), i.e. bacterial production (BP) per unit of assimilated carbon was calculated using equation 1.


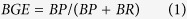


### Bacterial community analyses

Unfiltered water samples for DNA extraction were frozen within 2–4 hours of sampling. A subsample of 300 ml frozen water was freeze dried with an Alpha 1–4 LD plus (Christ, Osterode, Germany). DNA extraction, PCR (primers 341F (5′-CCTACGGGNGGCWGCAG-3′) and 805R (5′-GACTACHVGGGTATCTAATCC-3′)^58^) and 454-pyrosequencing were performed as described in Peura *et al.*^59^. The amplicon processing, including quality trimming and noise and chimera removal, was done as outlined in Schloss *et al.*^60^ using Mothur^61^. The sequences were assigned into operational taxonomic units (OTUs) using 97% sequence similarity cutoff, loosely corresponding to bacterial species and OTUs were classified using taxonomic framework for freshwater bacteria introduced by Newton *et al.*^62^. Two samples with likely fecal contamination were removed from the sample set. Prior to statistical testing, the sequence data was resampled to the smallest sample size (1153 sequences per sample) using perl script daisychopper.pl (available at http://www.genomics.ceh.ac.uk/GeneSwytch/Tools. Html^63^). The sequences are available at the NCBI Sequence Read Archive under project number PRNA244724.

### Statistical analysis

Differences in t-DOM variables and temperature-corrected bacterial metabolism among sites were tested using 2-way ANOVAs. Site and season were considered as fixed factors in the analysis. Normality and homogeneity of variance were checked with visual examination of residuals^64^. Square root transformations were applied to TN and Chl-a, logarithmic (base 10) transformations to a320, fulvic acids, BP and BR, and inverse (x^−1^) transformation to S289 to achieve ANOVA assumptions. When a factor was significant, a post hoc multiple comparison test (Tukey-Kramer) was carried out to identify differences. For the statistical testing of the BCC, all OTUs with more than 100 sequences in the resampled data were retained in the analysis. Pielou’s index was used to evaluate the evenness of the community in different sites, that is, how evenly the observations were distributed among OTUs^65^. To measure the diversity, we used inverse Simpson’s index^66^. Permutational Multivariate analysis of variance (PERMANOVA^67^) with 999 permutations was used to examine the impact of site to the bacterial community structure. Bacterial data were square root transformed prior to generating a resemblance matrix of Bray-Curtis similarities. Pairwise permutation *t*-tests were performed on the factors that were identified as significant in PERMANOVA to identify differences among sites. Multiple regression analyses were used to identify which DOM variables (TN, Chl-a, DOC, SUVA, S289, humic acids, fulvic acids and proteins) best explained the changes in bacterial metabolism (BP, BR, and BGE). The absorption coefficient a320 was omitted from the model due to its high Pearson correlation with DOC (r = 0.85) and humic acids (r = 0.96). TP was omitted due to its high correlation with TN (r = 0.88). Best model (using forward procedure) was selected according to the lowest value of Aikaike Information Criterion (AICc) index. Spearman’s rank correlations were used to examine relationships between the resemblance matrices of BCC and environmental variables to identify the environmental variables (alone or in subset) that best explain the observed patterns of BCC (BIO-ENV analyses, PRIMER). For this analysis, OTU and environmental variable matrices were constructed using Bray-Curtis dissimilarity (square-root transformed) and Euclidean distances respectively^68^,^69^. Diversity indices and relationships between BCC and carbon components were analysed with Spearman’s rank correlation in R^70^. The software JMP (JMP^®^, Version 10.0. SAS Institute Inc., Cary, NC, 1989–2012) was used for all univariate tests while PRIMER + PERMANOVA (version 6.1.6^67^,^71^) was used for multivariate analyses. A threshold of significance of 0.05 was adopted for all statistical tests.

## Results

### Expression of t-DOM in subarctic freshwaters

Many of the environmental variables had variation following the *a priori* assumption of differences in the t-DOM exposure among different sampling sites. Concentrations for DOC (F_2,29_ = 5.10, p = 0.0127), proteins (F_2,27_ = 9.87, p = 0.0006), total phosphorus (TP) (F_2,29_ = 12.88, p < 0.0001), and total nitrogen (TN) (F_2,29_ = 10.83, p = 0.0003), were significantly different among sites and highest in the ponds and lowest in the outlets (Table 1). Similarly, the total amount of coloured DOM (CDOM_320_) and the fluorescence of humic-like compounds (indicator of the share of terrestrial carbon in the CDOM) were lowest in the outlets, though this difference was not significant. S289, an indicator of algal carbon availability, was always significantly higher in the outlets (F_2,27_ = 50.17, p < 0.0001) than in the other sites while chlorophyll a (Chl-a), another indicator of algal carbon, was equally low in all samples (<1 μg L^−1^) (Table 1).

### Bacterial metabolism and community composition

**Figure 1:**
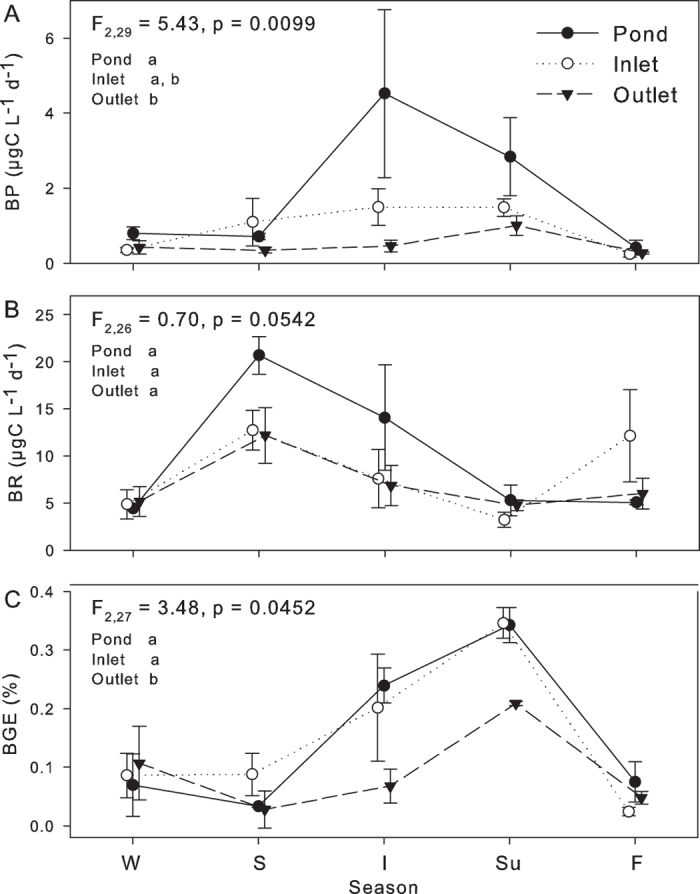
Mean yearly bacterial metabolism ± SE for ponds, inlets and outlets measured as (**a**) bacterial production (BP), (**b**) bacteria respiration (BR) and (**c**) bacterial growth efficiency (BGE). W = winter, S = spring, I = ice breakup, Su = summer and F = fall. The letters indicate statistical differences among sites.

The ponds and inlets provided an environment that supported higher BP and BGE than the outlets while there was less variation in BR among different sampling sites (Fig. 1). In all sites bacterial metabolism exhibited large seasonal variation (with all p-values < 0.05 for season factor) with some differences between the sites (Fig. 1). Highest BP values were measured for the ponds (4.5 μg C L^−1^ d^−1^ ± 3.9) and inlets (1.5 μg C L^−1^ d^−1^ ± 0.8) during the ice breakup while the maximum BP in the outlets (1.0 μg C L^−1^ d^−1^ ± 0.5) was reached in summer. The BP was lowest in the fall in all sites with values <1 μg C L^−1^ d^−1^. BR followed a different seasonal pattern, with the highest values measured in the ponds in the spring (20.7 μg C L^−1^ d^−1^ ± 4.4) and the lowest in the inlets in the summer (3.2 μg C L^−1^ d^−1^ ± 1.2). BGE was rather low and the maximum values, 20–39%, were reached in the summer.

**Figure 2:**
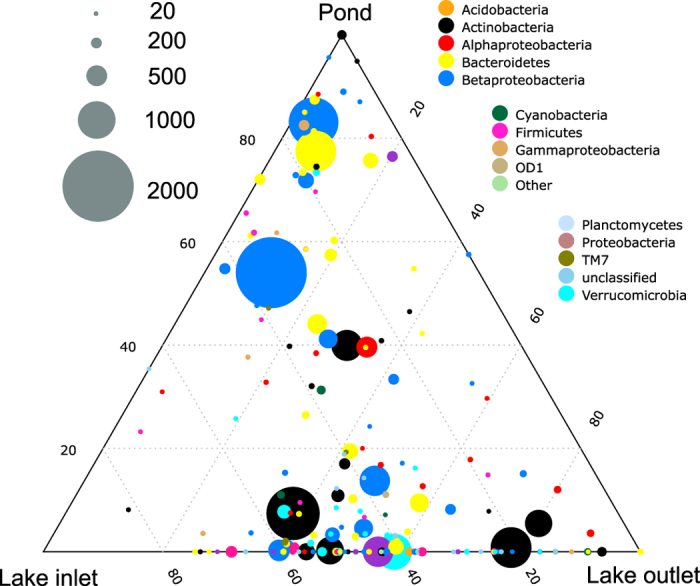
Ternary plot showing the distribution of OTUs between the habitats. Axes represent the pond, inlet and outlet and the percentage of reads associated with each environment. The size of the symbol indicates number of reads associated with each OTU and taxonomic affiliations are indicated by colors. All OTUs with at least 20 reads were included into the plot.

The sites also differed in bacterial community composition. The diversity in the inlets and outlets was higher (Inverse Simpson index) (χ^2^ = 11.97, p < 0.005) than in the ponds but there was no difference in the evenness (Pielou’s Index) of the communities across sites (Table 2). However, there was a clear change in the community structure among the sampled sites (Pseudo-F_2,22_ = 5.76, p < 0.001). The pond communities were more similar to the inlet communities (pair-wise test t = 1.77, p = 0.019) than to the outlet communities (t = 3.76, p < 0.001), but also the inlet and outlet communities were distinct from each other (t = 1.61, p = 0.037). The ponds and inlets had only a few, but very abundant OTUs, while the outlets harbored many low abundance OTUs (Fig. 2). There was also a separation in taxa distribution between ponds and lake habitats with taxa such as *Betaproteobacteria* (tribes PnecC (OTU 10973), Lhab-A2 (OTU10878)) and *Bacteroidetes* (clade bacIII-A (OTU 10854)) being more typical for ponds, while inlets and outlets had a higher abundance of *Actinobacteria* (tribe Myco (OTU 10771) and clade acI-A (OTU 10977)), *Verrucomicrobia* (OTU 10891) and *Alphaproteobacteria* (tribe LD12 (OTU 10100)) (Fig. 2).

### The control of subarctic freshwater microbiome by t-DOM

Multiple regression models were constructed to assess the importance of each DOM variable that was confirmed to have a significant impact on the BP, BR and BGE. The models explained up to 62% of the variance in BP, 87% in BR and 26% in BGE (Table 3). Overall, TN explained the largest share of the bacterial metabolism (on average 45%), but there was a lot of variation between sites and processes. The highest explanatory degree was acquired from the BR in ponds, where concentrations of TN and Chl-a explained 66 and 21% of the variation, respectively.

**Figure 3:**
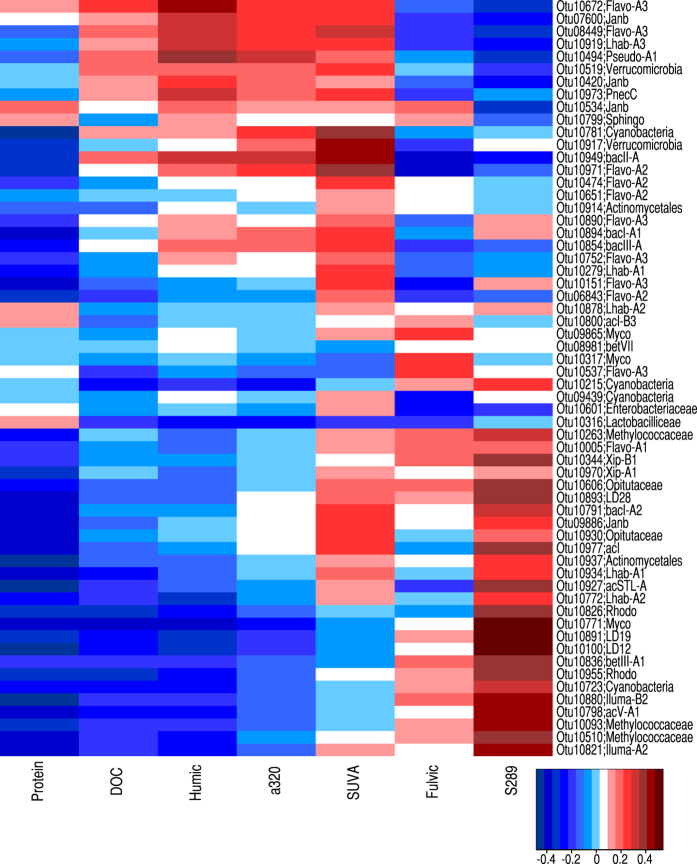
Heatmap visualizing the Spearman correlations between abundances of OTUs and concentrations of different fractions of CDOM.

The BIO-ENV analyses suggested that DOM variables that best explained the OTU distribution among sites were TP, DOC, fulvic acids and proteins (Table 4). The proteins represent readily available amino acid-like fraction of DOM and they were the carbon compounds that best captured most of the variability. The Spearman correlations further suggested connections between certain bacterial groups and carbon fractions (Fig. 3). For example, most OTUs associated with flavobacterial tribe Flavo-A3 were positively correlated with humic fraction and SUVA-index, both of which indicate the share of terrestrial DOC. Also all OTUs associated with betaproteobacterial tribe Janb had positive correlation with SUVA. S289, an indicator for algal carbon, had correlations for example to alphaproteobacterial lineage LD12, betaproteobacterial LD28 and verrucomicrobial LD19. The protein fraction appeared to favor only a few OTUs and all of the protein correlations were weak.

## Discussion

Microbiological properties of the sites reflected the large variations in DOM characteristics in subarctic freshwaters, ranging from sites heavily exposed to fresh terrestrial carbon inputs to sites only receiving processed, recalcitrant terrestrial DOM. Shallow ponds that were abundant in nutrients, t-DOM and algal exudates favoured high microbial productivity but supported low microbial diversity. At the same time the nutrient- and humic-poor lake outlets that were least exposed to t-DOM had low bacterial metabolism but a diverse community with high number of OTUs. The fact that t-DOM associated nutrients and carbon compounds were driving the metabolism and BCC indicate that ponds are hotspots in processing and sequestering terrestrial carbon. Considering the increase in number of ponds and loads of terrestrial carbon^9^,^10^,^11^ and our finding of lower bacterial diversity in ponds, changes in the GHG emissions to the atmosphere and in the biodiversity of the subarctic and arctic biomes may be expected.

Concentrations of DOC, different CDOM compounds and nutrients were highest in the ponds, most likely due to their high perimeter to volume ratio that facilitated high inputs of OM from the catchment^7^,^38^. The concentrations were especially elevated under the ice in winter and spring (on average 9.2 μg L^−1^ TP, 337 μg L^−1^ TN, 4.3 mg L^−1^ DOC and 0.8231 R.U. for humic compounds) when they were 19–42% higher than inlet values and 40–51% higher than in the outlets. The high under ice values likely resulted from a combination of microbial-derived dissolved compounds and out-freezing inputs associated with formation of ice. The latter one impacts especially small water bodies and may partly explain the higher values in ponds. Similar under ice accumulation of DOM in subarctic ponds and lakes has also been reported previously^7^,^72^. In other seasons the t-DOM and associated compounds were on average 8 and 19% higher in the ponds than in the inlets and outlets, respectively. In general, higher concentrations of compounds are typical for small subarctic water bodies due to a high perimeter to area ratio and lack of dilution effect of large lakes. Thus, the highest terrestrial impact to the carbon quality was in the ponds. In addition to terrestrial production, the humic fraction could also originate from *in situ* production by microbes^73^, but based on low values of S289 observed, it can be assumed that the contribution of fulvic and humic compounds from autochthonous production was minor^44^.

We used S289 and proteins as proxies of the aromatic content of CDOM and the relative importance of autochthonous (algal) carbon sources. Changes in spectral slope of DOM samples are due a number of processes, including photo and microbial degradation, mixing and production of low molecular weight CDOM. High values close to S289 have earlier been identified in different phytoplankton monocultures and used as an index of autochthonous DOM^44^,^45^,^46^. Consistent with our hypothesis and Jonsson *et al.*^74^, carbon in the lake outlets was characterized by highest values of S289 (annual average 0.0186 versus 0.0137 for ponds and 0.0158 for inlets) suggesting important algal contribution to the DOM pool in outlets. The measured lake outlet S289 values were also very close to the values recently measured for other subarctic lakes in the same region^46^. Also the fact that proteins were higher in the outlets than in the inlets supports strong algal impact on the outlet DOM, as the main producers of amino acids (proteins) in lakes is phytoplankton^25^,^75^. Thus, primary production was a major contributor to the DOM pool in the outlets. These labile fractions of CDOM showed seasonal variability in ponds where the algal exudates were low under ice according to the S289 values (0.0099 under the ice vs 0.0151 in summer). Proteins, however, were rich under the ice in ponds, most likely resulting from bacterial degradation of autochthonous material^15^,^76^ rather than from algal activity. We propose the same causality for the seasonally similar values in S289 and proteins in inlets and outlets. Hence, they likely resulted from a combination of algal leachates during the growing season and from the bacterial degradation of phytoplankton and other POM in winter, reflecting the 8 months of ice cover in the studied sites when degradation dominates over production^72^.

Bacteria production rates in open water period were in the lower range reported for subarctic and arctic ponds and lakes^77^, but with values similar to a few years earlier in the same region^35^. The water bodies in the studied area are also more nutrient-poor than most other subarctic lakes^78^, likely contributing to the lower BP rates^79^. Variation in the concentration of different carbon fractions across sites suggested that BP and BGE in ponds were supported by high concentrations of nutrients, humic acids and proteins. Accordingly, multiple linear regression models indicated that, in accordance with previous studies^80^,^81^,^82^, the strongest factor controlling bacterial metabolism was TN concentration. TP also likely controlled bacterial metabolism, however, due to its high correlation with TN (r = 0.88), TP was excluded from the model. Concentration and composition of organic carbon have also been suggested to be key factors regulating BGE and BP^35^,^83^,^84^. However, we did not see any link between DOC concentration and BP, but in contrast, the humic fraction of DOM had a positive impact on BP. Many compounds in the humic fraction of DOC are regarded as recalcitrant to bacterial degradation^26^ and reported to support lower BP than the non-humic fraction of DOC^85^. However, humic compounds are also highly sensitive to photodegradation^16^,^53^,^86^, which generates products that enhance bacterial metabolism^87^. The occurrence of humic-like substances was highest during the ice break up in June when also the intensity of solar radiation increased in the water column after the dark winter and was at its annual maximum. Thus, photodegradation of humic compounds likely contributed to the observed increase in BP, and more so in the shallow ponds and inlets than in the outlets. Further, some low molecular weight molecules of t-DOM have recently been shown to be highly reactive and to support high levels of bacterial metabolisms^2^.

Both, DOC source^30^,^31^,^88^ and quality^74^, have been shown to impact BCC. Also here the overall composition of bacterial community was controlled by concentration and composition of carbon as well as by nutrient concentrations. In ponds, where bacterial diversity was much lower than in other sites, carbon quality was different from the lake sites and one of the most important factors contributing to variation in BCC. We propose that the smaller bacterial diversity in the ponds results from a combination of carbon substrata dominated by t-DOM and the small size of ponds, which is known to negatively correlate with bacterial diversity^89^.

The combination of molecular microbiology and chemical analyses enabled us to link certain bacterial tribes to carbon fractions across habitats. Our environmental data corroborates experimental results suggesting that members of tribe Lhab have a preference to algal carbon over terrestrial carbon^32^. Another interesting link was seen between two indicators of terrestrial carbon (humic fraction and SUVA) and OTUs associated with flavobacterial tribe Flavo-A3. Bacteria associated with this group have previously been suggested to benefit from phytoplankton exudates^90^, which is opposite to what was observed here. However, in a review study 30% of the previous observations of tribe Flavo-A3 were from soil habitats^62^, suggesting that Flavo-A3 consists of at least two groups of bacteria with very distinct environmental preferences. Another group in the bacterial community that was associated with terrestrial carbon was tribe Janb. *Janthinobacterium*, the representative genus of tribe Janb, is described as soil bacterium^62^. Thus, both Flavo-A3 and Janb could be transient members in the lake community and may originate from the catchment area. There were also groups that were associated only with algal carbon. These included, for example, alphaproteobacterial tribe LD12. This tribe is a sister group of highly abundant marine cluster SAR11 and has been described as typical for freshwater habitats^91^. The previous reports suggest that the members of tribe LD12 are poor competitors and their abundance has previously been reported to be negatively correlated with phytoplankton^92^. However, it has also been shown that there is generally a lot of variation in substrate and environmental preferences within bacterial tribes^82^ and even within species^93^,^94^. Further, for LD12 specifically it has been suggested that this tribe has wide variations in environmental preferences across lakes^92^. Thus, it is not surprising that we see variation in preferences between the members of same tribe residing in sites that are differently exposed to t-DOM.

## Conclusions

Our study demonstrates how the variability of DOM in subarctic waters is tightly connected to the exposure of the site to terrestrial organic matter inputs. This creates variation beyond what is seen within habitat-specific studies and allowed a more detailed evaluation of the consequences that increased catchment-lake coupling, currently taking place across the circumpolar North, has for aquatic ecosystems. We show that the combination of terrestrial and algal derived carbon compounds and nutrients, such as the DOM in the ponds, supported the highest BP and BGE. Thus, considering the rising number of ponds, the importance of terrestrial carbon in fueling aquatic food webs is increasing in the subarctic and arctic region. We also showed that the microbial diversity is smaller in ponds and the community composition different from other subarctic inland waters. Small water volume, short water retention time, high seasonality and terrestrial impact all likely contribute to shaping the microbiome in the ponds. This all combined implies that the increasing number of terrestrially influenced ponds in the North will modify the quality of carbon that is recycled in freshwaters towards the terrestrial fraction and at the same time possibly reduce the aquatic microbial diversity. While the surface area of individual ponds is small, their contribution to greenhouse gas emission per surface area is higher than that of larger lakes^95^. Further, the high bacterial metabolism and terrestrial contribution in the ponds implies that in the subarctic and arctic region the contribution of heterotrophic systems increases and the importance of autotrophic systems decreases. This could mean that there may be an increase in the carbon flow from arctic waters to the atmosphere. Thus, these ecosystems should be acknowledged as hot spots of increasing importance in the carbon cycling of the arctic landscape.

## Additional Information

**How to cite this article**: Roiha, T. *et al.* Allochthonous carbon is a major regulator to bacterial growth and community composition in subarctic freshwaters. *Sci. Rep.* **6**, 34456; doi: 10.1038/srep34456 (2016).

## Supplementary Material

Supplementary Information

## Figures and Tables

**Table 1:** Mean values of temperature, total phosphorus (TP), total nitrogen (TN), chlorophyll-a (chl-a), dissolved organic carbon (DOC), specific UV-absorbance index (SUVA_254_), absorption at 320 nm (a_320_), spectral slope at 289 nm (S289) and fluorescence intensity of humic, fulvic and protein compounds of DOC in Raman units (R.U) for the three different water pools.

Site	Season	Temp (°C)	TP (μg L-1)	TN (μg L-1)	Chl-a (μg L-1)	DOC (mg L-1)	a320	SUVA254 (mgC L-1 m-1)	S289	Humic (R.U.)	Fulvic (R.U.)	Protein (R.U.)
Pond	W	0.1	8.0	350	0.51	3.9	19.8	3.8	0.0099	0.7031	0.0075	0.7342
Inlet	W	0.1	6.0	174	0.11	2.8	6.6	2.6	0.0173	0.6848	0.0562	0.2815
Outlet	W	0.3	5.3	132	0.09	2.3	4.7	2.4	0.0190	0.4871	0.0538	0.3247
Pond	S	0.1	10.0	381	0.22	4.6	9.1	1.7	0.0132	0.9431	0.0872	0.6349
Inlet	S	0.4	6.6	218	0.16	2.6	6.5	2.6	0.0156	0.6465	0.0660	0.3547
Outlet	S	0.5	5.7	198	0.19	2.2	4.9	2.4	0.0197	0.4904	0.0531	0.3384
Pond	I	12.0	6.7	144	0.18	2.8	6.9	2.5	0.0152	0.7339	0.1115	0.3966
Inlet	I	10.2	5.7	116	0.29	2.5	7.7	3.2	0.0151	0.8036	0.0344	0.2771
Outlet	I	6.3	5.7	137	0.34	2.3	5.9	2.7	0.0173	0.5803	0.0491	0.2783
Pond	Su	14.4	5.7	136	0.16	2.9	7.4	2.6	0.0154	0.8631	0.0736	0.3374
Inlet	Su	13.6	5.3	145	0.20	2.6	6.7	2.7	0.0155	0.7303	0.0339	0.2924
Outlet	Su	13.1	5.0	120	0.19	2.4	5.1	2.3	0.0185	0.4702	0.0702	0.3134
Pond	F	0.5	6.0	136	0.25	2.5	6.9	2.7	0.0147	0.6996	0.0354	0.2542
Inlet	F	2.4	5.7	139	0.22	2.0	5.8	3.0	0.0155	0.6121	0.0588	0.3253
Outlet	F	4.9	5.0	121	0.54	2.2	6.2	2.4	0.0187	0.4333	0.0569	0.2652
Pond	All	5.4 a	7.3 a	229 a	0.27 a	3.2 a	10.0 a	2.7 a	0.0137 a	0.7886 a	0.0630 a	0.4715 a
Inlet	All	5.4 a	5.9 b	158 b	0.18 a	2.4 ab	6.7 a	2.7 a	0.0158 ab	0.6955 a	0.0499 a	0.3062 b
Outlet	All	5.0 a	5.3 b	142 b	0.27 a	2.3 b	5.4 a	2.4 a	0.0186 b	0.2461 a	0.0566 a	0.3040 b

Data are shown for five seasons in 2011: winter (W), spring (S), ice break (I), summer (Su), and fall (F) as well as for the entire year (All). The letters (for All) indicate statistical differences between sites.

**Table 2:** Shannon and Inverse Simpson indices (±SE) for different sites.

Site	Inverse Simpson	Pielous Index
Pond	6.2 ± 4.2	0.45 ± 0.07
Inlet	16.4 ± 10.0	0.47 ± 0.07
Outlet	18.6 ± 7.9	0.49 ± 0.03

**Table 3:** Results of different multiple linear regression models (based on lowest AICc) to estimate a) bacterial production (BP), b) bacteria respiration (BR) and c) bacteria growth efficiency (BGE) for all data and for ponds, inlets and outlets separately.

	Intercept	Humic	TN	S289	Chl-a	N	R^2^ (adj. R^2^)	AICc	RMSE
a) BP									
*All data*	−1.00	0.86	0.24	ns	ns	42	0.26 (0.22)	105.65	0.816
Partial R^2^		0.07	0.19						
*Pond*	—	ns	ns	ns	ns	13	—	—	—
Partial R^2^									
*Inlet*	4.77	ns	0.007	−320.31	ns	15	0.62 (0.56)	30.12	0.495
Partial R^2^			0.38	0.24					
*Outlet*	—	ns	ns	ns	ns	14	—	—	—
Partial R^2^									
b) BR									
*All data*	−2.45	ns	0.078	ns	ns	39	0.36 (0.22)	105.65	0.816
Partial R^2^			0.36						
*Pond*	−4.80	ns	0.12	ns	−24.77	13	0.87 (0.84)	80.23	4.47
Partial R^2^			0.66		0.21				
*Inlet*	—	ns	ns	ns	ns	14	—	—	—
Partial R^2^									
*Outlet*	−7.16	ns	0.11	ns	ns	13	0.80 (0.79)	75.67	3.46
Partial R^2^			0.80						
c) BGE									
*All data*	0.63	ns	−00006	−22.10	−0.18	39	0.26 (0.20)	−52.48	0.11
Partial R^2^			0.07	0.11	0.08				
*Pond*	—	ns	ns	ns	ns	13	—	—	—
Partial R^2^									
*Inlet*	—	ns	ns	ns	ns	14	—	—	—
Partial R^2^									
*Outlet*	—	ns	ns	ns	ns	13	—	—	—
Partial R^2^									

Humic acids (Humic), total nitrogen (TN), spectral slope at 289 nm (S289) and chlorohyll-a (Chl-a) were the variables used in the regression models (only significant values are listed). ns: not significant. Partial R^2^ below each regression coefficient, N = number of data included, total R^2^ (adjusted R^2^), small sample size–corrected AICc Index and root mean square errors (RMSE) are shown.

**Table 4:** Combinations of environmental variables (TP, TN, DOC, Chl-a, S289, SUVA, humic, fulvic and protein), taken *k* at a time, giving the four best variables alone and the largest rank correlation *ρ*_*s*_ between OTU and environmental variable similarity matrices; bold indicates the best combination overall.

k	Best variable combinations (*ρ*_*s*_)			
1	Protein (0.42)	DOC (0.38)	TN (0.35)	TP (0.34)
3	TP, fulvic, protein (0.54)			
4	**TP, DOC, fulvic, protein (0.57)**	TP, humic, fulvic, protein (0.55)	TP, S289, fulvic, protein (0.54)	
5	TP, DOC, S289, fulvic, protein (0.56)	TP, DOC, humic, fulvic, protein (0.56)	TP, DOC, SUVA, fulvic, protein (0.55)	TP, S289, humic, fulvic, protein (0.57)
