# Supplementary material for: Allochthonous carbon is a major regulator to bacterial growth and community composition in subarctic freshwaters
Source: Sci Rep. 2016 Sep 30;6:34456. doi: 10.1038/srep34456 (PMC5043279; doi:10.1038/srep34456)
Supplement: Supplementary Information [file srep34456-s1.doc]

Allochthonous carbon is a major regulator to bacterial growth and community composition in subarctic freshwaters

# Toni Roiha^1,2†^, Sari Peura^1,3*†^, Mathieu Cusson^2^ and Milla Rautio^1,2,4^

Supplementary Table 1. Characteristics of the sampled ponds (11, 12, 15), lakes (outlets) and rivers (inlets). Depth refers to the maximum depth of the site. River lengths are calculated only for the section of the river between the inlet sampling point and the next lake upstream. Nd = no data.

| Site | Latitude  (N) | Longitude  (E) | Altitude (m a.s.l.) | Catchment area (ha) | Lake area (ha) | River length (m) | Depth (m) | DOC (mg l^-1^) | Tot N (µg l^-1^) | Tot P (µg l^-1^) |
| --- | --- | --- | --- | --- | --- | --- | --- | --- | --- | --- |
| Pond 11 | 69° 3.584' | 20° 51.056' | 710 | 45 | 0.8 | - | 2 | 6.6 ±5.3 | 304 ±293 | 8.0 ±2.3 |
| Pond 12 | 69° 3.491' | 20° 51.336' | 710 | 54 | 1.3 | - | 3 | 2.7 ±0.8 | 200 ±97 | 7.0 ±1.4 |
| Pond 15 | 69° 3.003' | 20° 49.570' | 850 | 28 | 0.6 | - | 7 | 2.8 ±2.0 | 195 ±139 | 6.5 ±2.4 |
| Inlet Kilpisjärvi | 69° 0.803' | 20° 52.162' | 472 | nd | - | 2100 | 2 | 3.0 ±0.4 | 183 ±62 | 6.2 ±1.1 |
| Inlet Saanajärvi | 69° 3.282' | 20° 51.739' | 679 | nd | - | 1200 | 1 | 2.2 ±0.5 | 147 ±50 | 6.3 ±1.0 |
| Inlet Tsahkaljärvi | 69° 1.765' | 20° 53.673' | 560 | nd | - | 4200 | 2 | 2.3 ±0.7 | 124 ±30 | 5.0 ±0.0 |
| Outlet Kilpisjärvi | 68° 56.406' | 20° 51.320' | 472 | 26 000 | 3700 | - | 57 | 2.0 ±0.2 | 167 ±92 | 5.4 ±0.9 |
| Outlet Saanajärvi | 69° 2.58' | 20° 52.594' | 679 | 461 | 70 | - | 24 | 1.8 ±0.1 | 119 ±6 | 5.0 ±0.0 |
| Outlet Tsahkaljärvi | 69° 1.233' | 20° 54.032' | 560 | 3396 | 113 | - | 25 | 3.0 ±0.2 | 138 ±24 | 5.6 ±0.9 |
